# Supplementary material for: A universal metasurface antenna to manipulate all fundamental characteristics of electromagnetic waves
Source: Nat Commun. 2023 Aug 24;14:5155. doi: 10.1038/s41467-023-40717-9 (PMC10449906; doi:10.1038/s41467-023-40717-9)
Supplement: Supplementary file 1 — Supplementary Information [file 41467_2023_40717_MOESM1_ESM.pdf]

Supporting Information for

A universal metasurface antenna to manipulate all fundamental characteristics of electromagnetic waves

Geng-Bo Wu[1,2†], Jun Yan Dai[3,4,5†,*], Kam Man Shum[1], Ka Fai Chan[1], Qiang Cheng[3,4,5*], Tie Jun Cui[3,4,5*] and Chi Hou Chan[1,2,6*]

[1]*State Key Laboratory of Terahertz and Millimeter Waves, City University of Hong Kong, Hong Kong, 999077, China*

[2]*Department of Electrical Engineering, City University of Hong Kong, Hong Kong, 999077, China*

[3]*State Key Laboratory of Millimeter Waves, Southeast University, Nanjing 210096, China*

[4]*Institute of Electromagnetic Space, Southeast University, Nanjing 210096, China*

[5] *Frontiers Science Center for Mobile Information Communication and Security, Southeast University, Nanjing 210096, China*

[6]*Guangdong-Hong Kong Joint Laboratory for Big Data Imaging and Communication, Shenzhen 518048, China*

[†]These authors contributed equally to this work

[*]Corresponding author. Email: junyand@seu.edu.cn, qiangcheng@seu.edu.cn, tjcui@seu.edu.cn, eechic@cityu.edu.hk

This supplementary information contains the following sections:

Supplementary Note 1: Theoretical radiation pattern of the UMA

Supplementary Note 2: Derivation of equation (10) in the main text

Supplementary Note 3: Effects of the space envelope shift on the far-field pattern

Supplementary Note 4: Extension to a 2-D UMA

Supplementary Note 5: Study on coupling effects of the two ±45°-inclined slot openings

Supplementary Note 6: Effects of the 1D/2D aperture and designed focused distance on the near-field focused performance

Supplementary Table S1: Theoretical and measured $|u\rangle$ and $|v\rangle$ components for the six representative polarizations

Supplementary References

Supplementary Note 1: Theoretical radiation pattern of the UMA

Here, we leverage the discrete dipole approach to calculate the far-field radiation pattern of the universal metasurface antenna. The magnetic dipole moment is used as the weight coefficient in array factor calculation. Each meta-atom can be viewed as two ±45°-inclined magnetic dipoles, whose radiating far-field can be written as

$$\bar{\bar{H}}_i(\theta) = -\frac{\pi f^2}{r}\bar{\bar{m}}_i cos\theta e^{j\xi_m x_i sin\theta} \tag{S1}$$

where θ is the observation direction with respect to the surface normal of the metasurface. The pattern of the meta-atom is modeled as a cosine function $cos\theta$. Substituting equations (8) and (11) into (S1), one obtains

$$\bar{\bar{H}}_i(\theta) = -\frac{\pi H_0 P_0}{r}\sum_{m=-\infty}^{\infty} e^{j2\pi(f_0+mf_M)t} \cdot$$

$$(f_0 + f_M)^2 cos\theta\bar{\bar{\tau}}_i sinc(\pi m\bar{\bar{\tau}}_i)e^{-j(\xi_{gw}x_i - \xi_m x_i sin\theta + 2\pi m f_M \bar{\bar{t}}_i)} \tag{S2}$$

The far-field radiation pattern of the universal metasurface can be obtained by superposing the radiating fields from all the meta-atoms

$$\bar{\bar{H}}(\theta) = \sum_i^I A_i(\theta)AF$$

$$= -\frac{\pi H_0 P_0}{r}\sum_{m=-\infty}^{\infty} e^{j2\pi(f_0+mf_M)t}\sum_{i=1}^{I}(f_0 + f_M)^2 \cdot$$

$$cos\theta\bar{\bar{\tau}}_i sinc(\pi m\bar{\bar{\tau}}_i)e^{-j(\xi_{gw}x_i - \xi_m x_i sin\theta + 2\pi m f_M \bar{\bar{t}}_i)} \tag{S3}$$

Suppose the time gradient $\partial\bar{\bar{t}}_i/\partial x$ is a constant with $\bar{\bar{k}}_m = 2\pi m f_M\, \partial\bar{\bar{t}}_i/\partial x$, equation (S3) can be further written as

$$\bar{\bar{H}}(\theta, f + mf_M) = -\frac{\pi H_0 P_0}{r}\sum_{m=-\infty}^{\infty} e^{j2\pi(f_0+mf_M)t}\sum_{i=1}^{I}(f_0 + f_M)^2 \cdot$$

$$cos\theta\bar{\bar{\tau}}_i sinc(\pi m\bar{\bar{\tau}}_i)e^{-j(\xi_{gw} + \bar{\bar{k}}_m - \xi_m sin\theta)x_i} \tag{S4}$$

The beam radiation direction (output angle) of the PW is the direction where the extracted waves from all the meta-atoms interfere constructively as

$$\bar{\bar{\theta}}_r(f + mf_M) = sin^{-1}(\frac{\xi_{gw}+\bar{\bar{k}}_m}{\xi_m}) \tag{S5}$$

The extracted PW has a collimated beam in free space only when the momentum-matching condition $-\xi_m < \xi_{gw} + \bar{\bar{k}}_m < \xi_m$ is satisfied. Since different values of momentum $\bar{\bar{k}}_m$ are introduced to different harmonic frequencies, the space-time sequence was designed such that only the harmonic frequency of interest fulfills the momentum-matching condition in free space. The beam directions of other unwanted harmonics are out of the visible region; hence, these harmonics do not contribute to the radiation in free space.

Supplementary Note 2: Derivation of equation (10) in the main text

The equivalent magnetic polarizability $\bar{\bar{p}}_{i,m}$ for the ith meta-atom at the mth harmonic frequency in equation (10) are derived as follows:

$$\bar{\bar{p}}_{i,m} = \frac{1}{T_M}\int_0^{T_M} \bar{\bar{P}}_i(t) e^{-j2\pi m f_M t} dt$$

$$= P_0 \frac{1}{T_M}\int_{-\frac{T_M\bar{\bar{\tau}}_i}{2}}^{\frac{T_M\bar{\bar{\tau}}_i}{2}} e^{-j2\pi m f_M t} dt$$

$$= P_0 \frac{1}{T_M} \frac{e^{-j2\pi m f_M t}\Big|_{-\frac{T_M\bar{\bar{\tau}}_i}{2}}^{\frac{T_M\bar{\bar{\tau}}_i}{2}}}{-j2\pi m f_M}$$

$$= P_0 \bar{\bar{\tau}}_i sinc(\pi m \bar{\bar{\tau}}_i) \quad \text{(S6)}$$

Supplementary Note 3: Effects of the space envelope shift on the far-field pattern

The aperture field distribution $E(x)$ and its spatial frequency spectrum (i.e., far-field radiation pattern in free space) $F(\theta)$ is a Fourier transform pair

$$E(x) = A(x)e^{-j\xi_{gw}x} \xrightarrow{FT} F(\theta) \tag{S7}$$

where $A(x)$ is the equivalent sinusoidal amplitude envelope imparted by the spatiotemporal modulation at the fundamental frequency. According to the space-shifting property of the Fourier Transform, an analogy to the time-shifting property but in the space domain, we have

$$E(x - \Delta x) = A(x - \Delta x)e^{-j\xi_{gw}(x-\Delta x)} \xrightarrow{FT} e^{j\xi_0 sin\theta\Delta x}F(\theta) \tag{S8}$$

Now we introduce a space shift Δx to the spatial amplitude envelope. The aperture field of the metasurface antenna becomes $E'(x) = A(x - \Delta x)e^{-j\xi_{gw}x}$. Note that the spatial shift of the amplitude envelope does not affect the phase of the propagating guided wave $e^{-j\xi_{gw}x}$. Combining with equation (S8), we obtain

$$E'(x) = A(x - \Delta x)e^{-j\xi_{gw}(x-\Delta x)}e^{-j\xi_{gw}\Delta x}$$

$$\xrightarrow{FT} F'(\theta) = e^{-j(\xi_{gw}-\xi_0 sin\theta)\Delta x}F(\theta) \tag{S9}$$

We can observe from equation (S9) that a space translation Δx in the amplitude envelope introduces a direction-dependent phase shift $\Delta\phi(\theta) = -(\xi_{gw} - \xi_0 sin\theta)\Delta x$ to the radiation pattern.

Supplementary Note 4: Extension to a 2-D UMA

One solution for extending to a 2-D metasurface antenna is by periodically repeating the 1-D metasurface antenna along the y-axis, fed by a power-dividing network, as shown in Supplementary Fig. S10a. In this design, eight identical 1-D SIWs arrays with a total of 328 meta-atoms and 1312 PIN diodes form a 2-D radiating aperture. A similar topology for SIW-based 2-D antennas has been demonstrated in refs. [1,2], although without spatiotemporal modulation. It is important to point out that the array distance along the y-axis is not necessary to be subwavelength since the guided waves in each SIW waveguide only propagate along the x-axis. In this design, the array distance along the y-axis is 6.5 mm (around $0.5\lambda_0$ at 23.5 GHz). Thereby, the required PIN diode number for the 2D aperture can be significantly reduced.

Supplementary Fig. S10b shows the configuration of the 8-way SIW parallel feeding divider and its simulated E-field distribution at 23.5 GHz using a commercially available ANSYS HFSS numerical simulator. We can observe that the treelike power divider can divide the input power into eight parallel ways with the same amplitude and phase to feed the metasurface antenna array.

The 2D metasurface antenna can realize 2D wavefront engineering by extending the original 2D STC matrix into a 3D one. We demonstrate the 2-D beam scanning for the 1st harmonic radiation without loss of generality. Firstly, each 1-D metasurface antenna has its 2-D STC matrix to control the wavefront along the x-direction. We have demonstrated in Fig. 1e of the main text that the reference time shift can control the phase of the radiated wave for the 1-D metasurface antenna. To enable beam steering also in the y-direction, we apply different reference time shifts to the eight 1-D metasurface antennas to form a progressive phase shift along the y-direction. The corresponding eight different 2-D STC matrixes for the eight 1-D metasurface antennas for beam steering to $(\theta, \varphi) = (30°, 45°)$ are shown in Supplementary Fig. S11a. Cascading these eight 2D

STC matrixes forms a 3D STC matrix, as shown in Supplementary Fig. S11b. Supplementary Fig. S11c shows the corresponding theoretical radiation patterns in *uv*-space at different harmonic frequencies. The 3D radiation pattern at the 1st harmonic frequency is presented in Supplementary Fig. R11d. The radiation patterns are calculated based on the theoretical model for STC metasurface antennas[3]. We extend the 1-D spatial Fourier transform in Eq. (10) in ref. [3] to the 2-D spatial Fourier transform to calculate the 3-D radiation pattern here. We can observe from Supplementary Fig. S11c that a high-directivity beam radiates to the desired direction at the 1st harmonic frequency. Again, other undesired harmonics are highly suppressed due to the phase mismatch in each SIW waveguide and free space.

As proof-of-concept examples, the 2-D UMA is designed to steer the beam to $(\theta, \varphi) = (30°, 45°)$, $(0°, 0°)$ and $(30°, -135°)$, respectively. The theoretically calculated 3D radiation patterns at the 1st harmonic frequency are shown in Supplementary Fig. S11d-f. We can observe that the output beams of the 2D metasurface antenna can be correctly pointed in the intended directions. These results verify the 2D wavefront engineering of the 2D metasurface antenna.

Supplementary Note 5: Study on coupling effects of the two ±45°-inclined slot openings

To investigate the coupling effects of the two ±45°-inclined slot openings in each meta-atom, Supplementary Figs. S12a, b show two meta-atom configurations with and without the coupling from the other slot-opening. Meta-atom I with only one +45°-inclined slot-opening on the top of the SIW waveguide; Meta-atom II with two ±45°-inclined slot-openings, which is the configuration used in the main text and the experimental demonstration. In both cases, the PIN diodes in the +45°-inclined slot-opening are in the OFF state such that the meta-atom radiates $|v\rangle$ (-45° from the x-axis) linear polarization. In Meta-atom II, the PIN diodes in the -45°-inclined slot-opening are in the ON state. Supplementary Fig. S12d shows the two cases' simulated co- and cross-polarized radiation patterns using the full-wave simulator ANSYS HFSS. It can be observed that the co-polarized patterns are almost identical, while the cross-polarization level in the broadside direction ($\theta = 0^{\circ}$) increases from -20.9 dB to -17.9 dB for Meta-atom II with the existence of the -45°-inclined slot opening. This is due to the fact that the -45°-inclined slot-opening still contributes to the cross-polarization radiation, although the PIN diodes are in the ON state. In the theoretical model developed in the main text, the weak spatiotemporal coupling between the two slot openings is not considered, causing the deviation from the measurement results.

The coupling between the two slot openings can be significantly suppressed by shifting the resonant frequency of the -45°-inclined slot opening far away from the operating frequency. One solution is to adopt four PIN diodes at each slot opening, as shown in Meta-atom III in Supplementary Fig. S12c. The equivalent magnetic current path of the -45°-inclined slot opening is reduced, shifting the resonant frequency to a higher frequency than that of Meta-atom II. Supplementary Fig. S12d shows the simulated radiation patterns of Meta-atom III. We can observe

that the cross-polarization is similar to that of Meta-atom I, indicating the coupling from the -45°-inclined slot opening is negligible for Meta-atom III.

Supplementary Note 6: Effects of the 1D/2D aperture and designed focused distance on the near-field focused performance

To investigate the 1-D and 2-D antenna aperture effect on the fading distance along the z-direction, Supplementary Figs. S14a, d show the aperture phase distributions for the 1-D and 2-D antennas with an intended focus spot (x_F, y_F, z_F) = (0, 0, 80) mm, respectively. The radiated E-field distributions on the longitudinal (*xz*-plane) and transversal (*xy*-plane) planes calculated by the Fresnel diffraction approach[4] assuming a uniform aperture amplitude distribution are shown in Supplementary Fig. S14. From Supplementary Figs. S14c,f, we can observe that the 1-D antenna has a much broader beamwidth along the y-direction than that of the 2-D antenna since there is only one meta-atom along the y-direction. Nevertheless, the 1-D and 2-D aperture antennas share a similar field distribution in the longitudinal plane (*xz*-plane). As shown in Supplementary Figs. S14b,e, both the beams fade away at around 100 mm. Therefore, the 1-D radiating aperture only affects the field distribution along the *y*-direction, with negligible effects on the fading distance along the *z*-direction.

For the focused beam, the desired focused point determines the fading distance. Supplementary Fig. S15 shows the measured E-field intensity distributions for three different focus spot positions $F(x_F, y_F, z_F)$ = (0, 0, 40) mm, (0, 0, 60) mm, and (0, 0, 80) mm. It can be observed that a smaller depth of focus with a clear visual focusing effect is achieved as the designed focal point is closer to the universal metasurface antenna. The focusing effect is weaker as the designed focus moves away from the radiating aperture. In fact, the near-field focused effect disappears as the designed focal point shifts to infinite; in this case, the near-field focused antenna degenerates into far-field high-directivity antenna with an equal aperture phase distribution.

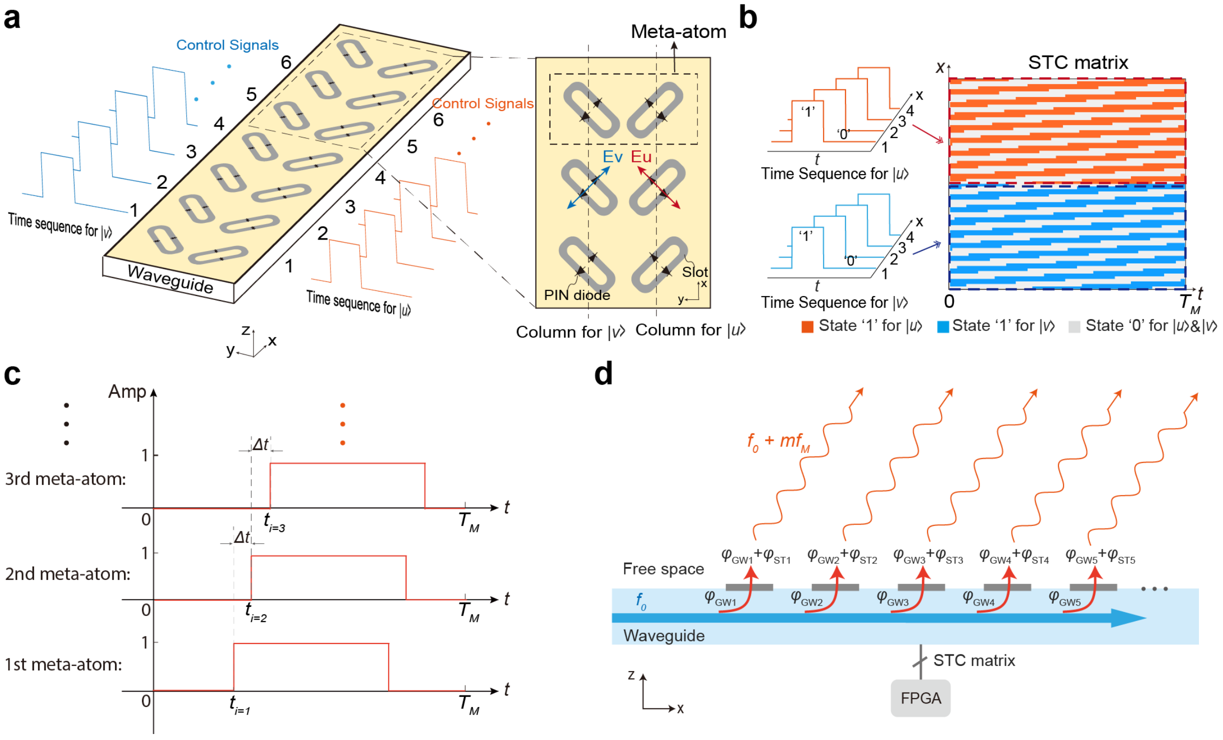

Supplementary Fig. S1 | The UMA for wave manipulations with frequency shifting. a, Configurations of the UMA, which consists of a meta-atom array located on the top of the waveguide. Each meta-atom consists of two ±45°-inclined slop openings to radiate two orthogonal eigen-polarization $|u\rangle$ and $|v\rangle$ states. PIN diodes, controlled by the external control signals from an FGPA, are embedded into each slot opening to switch the meta-atom between the radiating (coding element '1') and non-radiating (coding element '0') states in real time. **b,** The formulation of the '0/1' STC matrix, representing the radiating/non-radiating states of all the meta-atoms at one modulation period. Each inclined slot opening has its own independent time-coding sequence. The STC matrix consists of two parts, where the upper and lower parts correspond to slot-opening columns for $|u\rangle$ and $|v\rangle$ polarization radiations, respectively. **c,** Square time sequence for each meta-atom with a time shift t_i. **d,** Operating principle of the UMA for wavefront manipulation at harmonic frequencies. The total phase shift of the extracted wave consists of two parts: the phase accumulation due to the propagation of the guided wave $\varphi_{GW} = -\xi_{GW}x_i$, and the abrupt phase shift induced by the spatiotemporal modulation $\varphi_{ST} = -2\pi m f_M t_i(x)$. The time shifts applied to the meta-atoms can be utilized to control the momentum and phase at the target harmonic frequency.

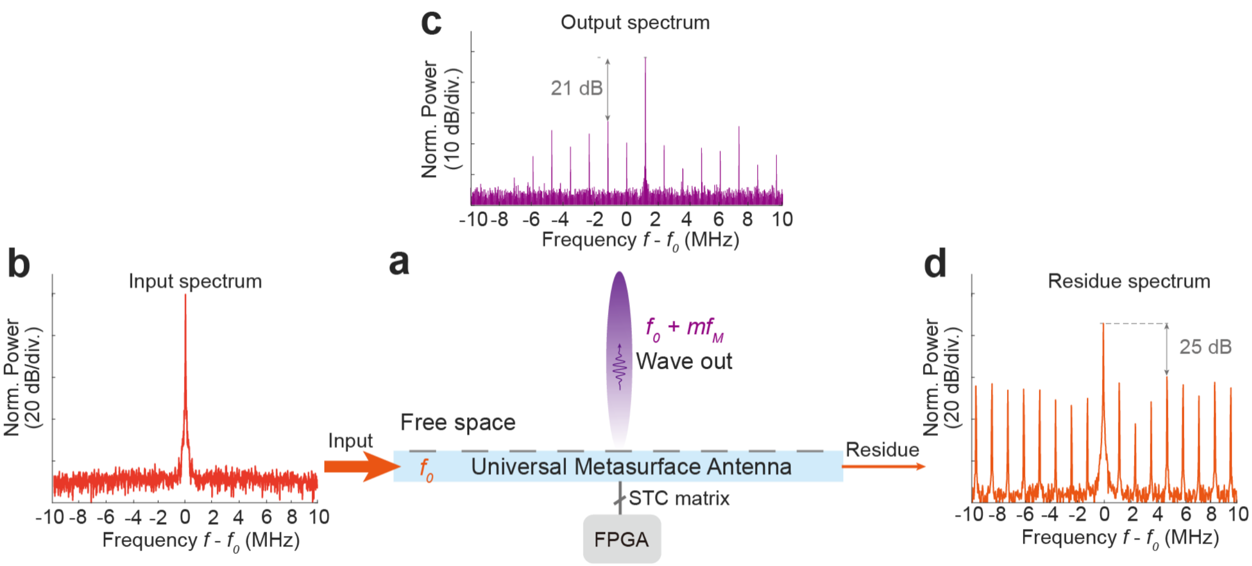

Supplementary Fig. S2 | The UMA for frequency shifting. a, The schematic diagram of the UMA for frequency shifting. In this illustrative example, the UMA upconverts the input frequency f_0 to $f_0 + f_M$ in free space. The input frequency and modulation frequency are $f_0 = 23.5$ GHz and $f_M = 1.2$ MHz, respectively. **b-d,** Measured input (**b**) and residual (**d**) spectra of the waveguide and the output spectrum in free space (**c**). The undesired harmonic frequencies inside the waveguide and free space are suppressed by as much as 25 dB and 21 dB below the fundamental frequency and target harmonic frequency, respectively, due to the large momentum mismatch inside the waveguide and free space. The residual power of the waveguide at the fundamental frequency is 14.7 dB below the input one due to the GW-to-PW conversion of the metasurface antenna.

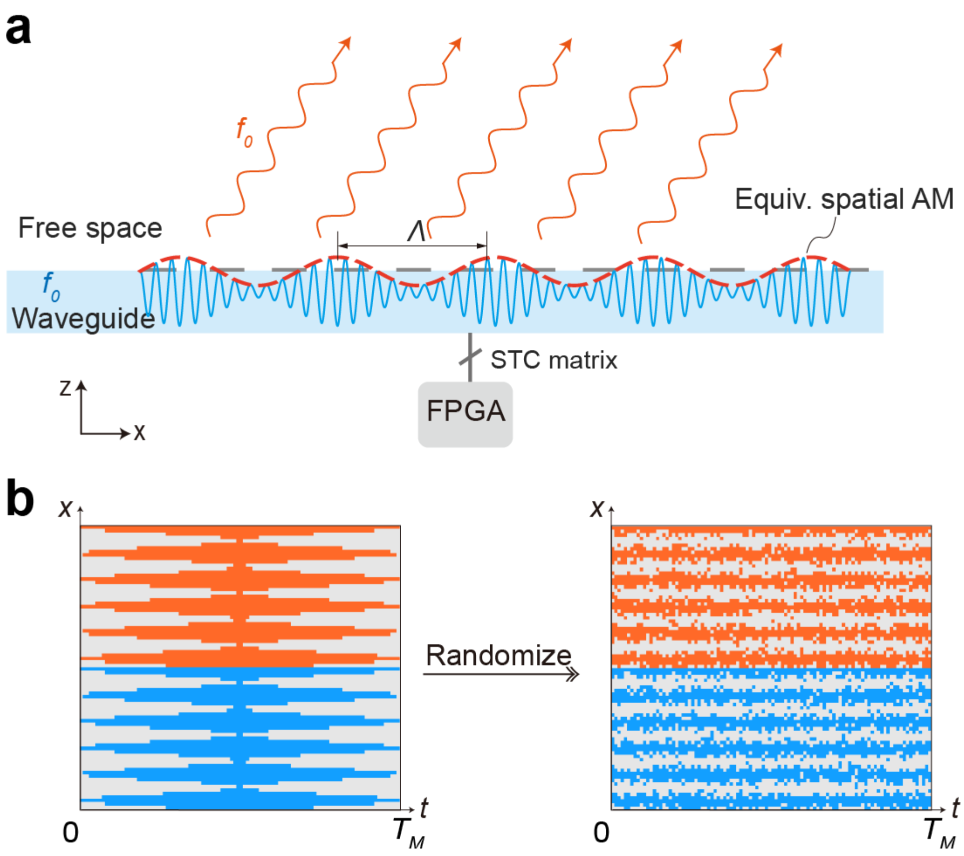

Supplementary Fig. S3 | The UMA for wave manipulation without frequency shifting. a, Schematic of the GW-to-PW translation at the fundamental frequency ($m = 0$). Spatiotemporal modulation forms an equivalent sinusoidal amplitude envelope along the length of the metasurface aperture. The $n = -1$ space harmonic becomes fast and radiates into free space with arbitrary software-defined wave properties. **b,** Randomization of the STC matrix to suppress higher-order harmonic frequencies.

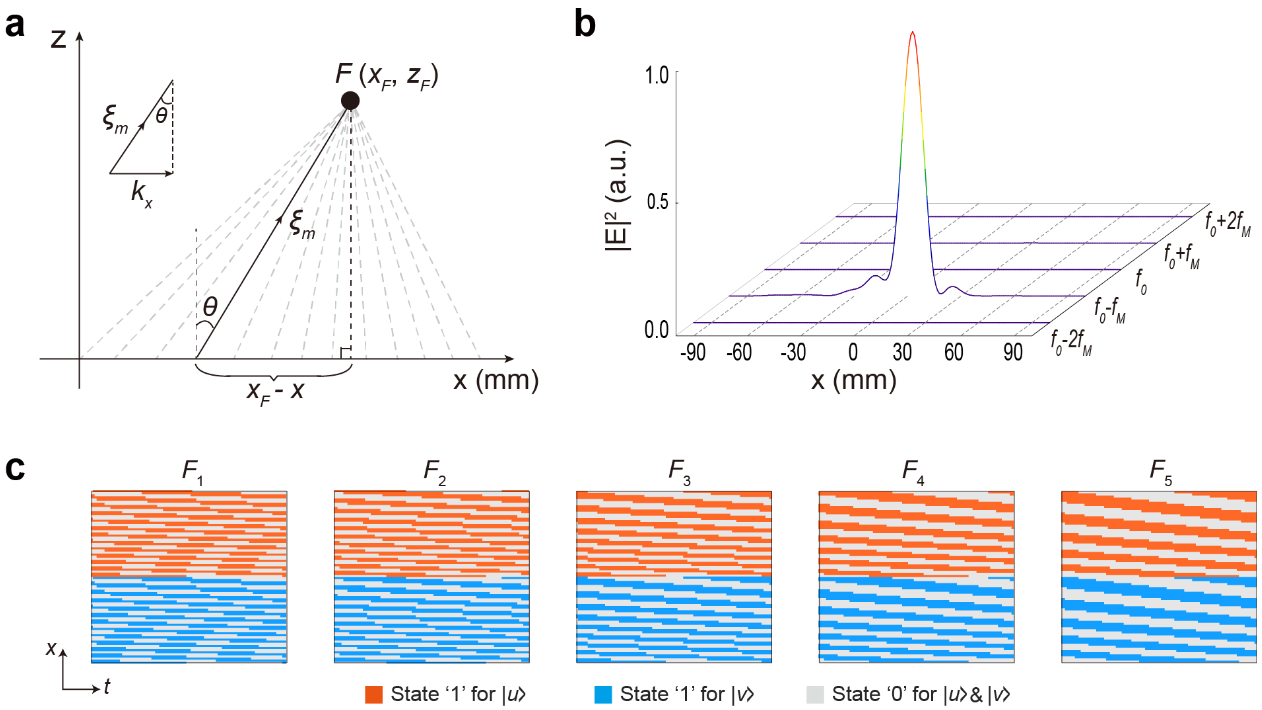

Supplementary Fig. S4 | More results on wave-focusing for the UMA. a, Geometric representation of the UMA to focus the extracted wave into a desired focal point $F = (x_F, z_F)$. **b,** Measured E-field density along the line z = 80 mm at different harmonic frequencies. High radiated power is observed only at the desired harmonic frequency $f_0 - f_M$, whereas other harmonic frequencies are highly suppressed. **c,** The STC matrixes applied to the UMA to focus the extracted wave at positions from F_1 to F_5.

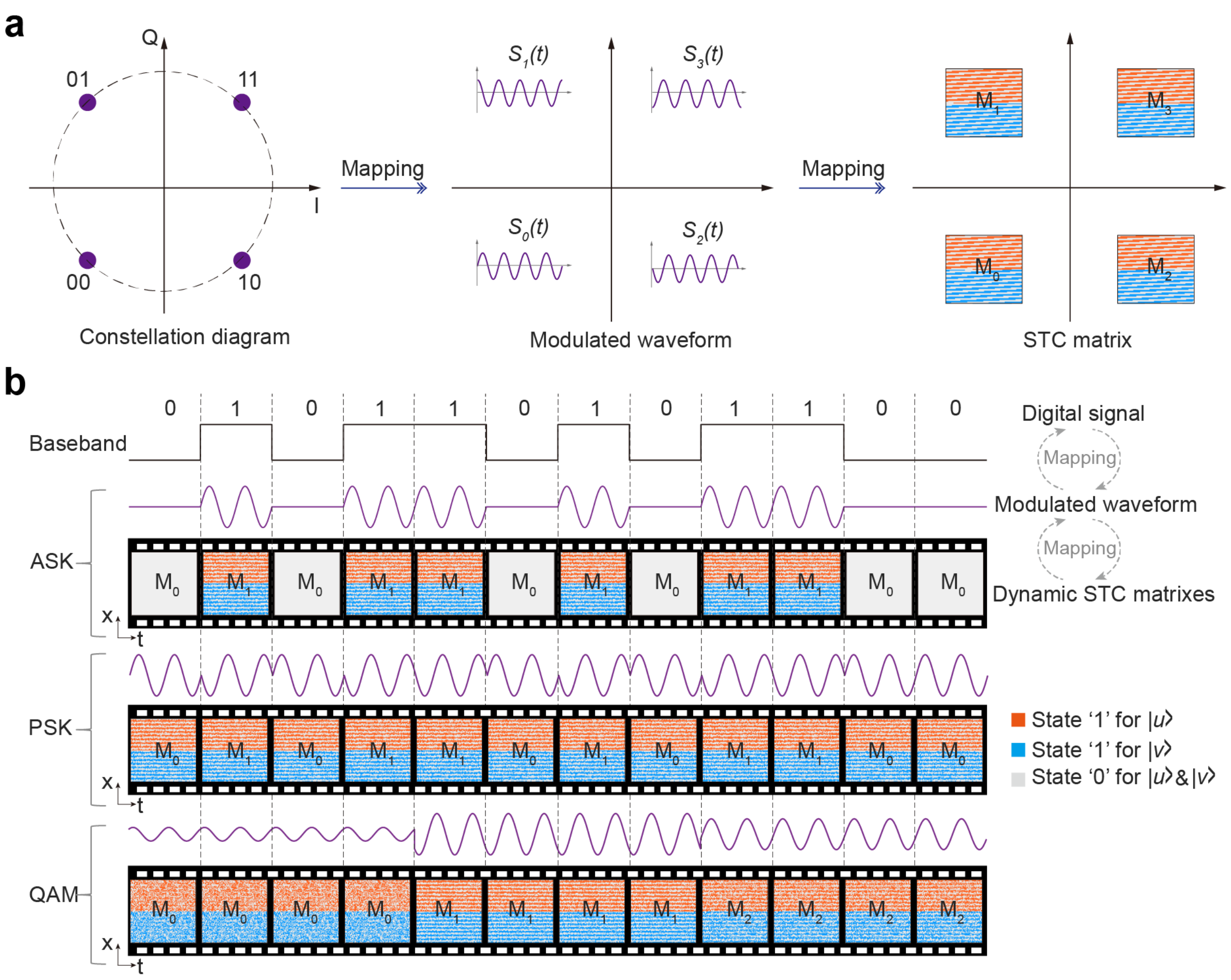

Supplementary Fig. S5 | The UMA for information manipulations with time-varying wave properties. a, The one-to-one mapping relationship among the QPSK symbol set in the constellation diagram, transmitted signal set, and STC matrix set ($m = +1$ harmonic frequency radiation in this case). **b,** The digital baseband signals, the corresponding modulated free-space waveforms, and the required dynamic STC matrixes (at the fundamental frequency in this case) for different modulation formats, including 2ASK, BPSK, and 16QAM.

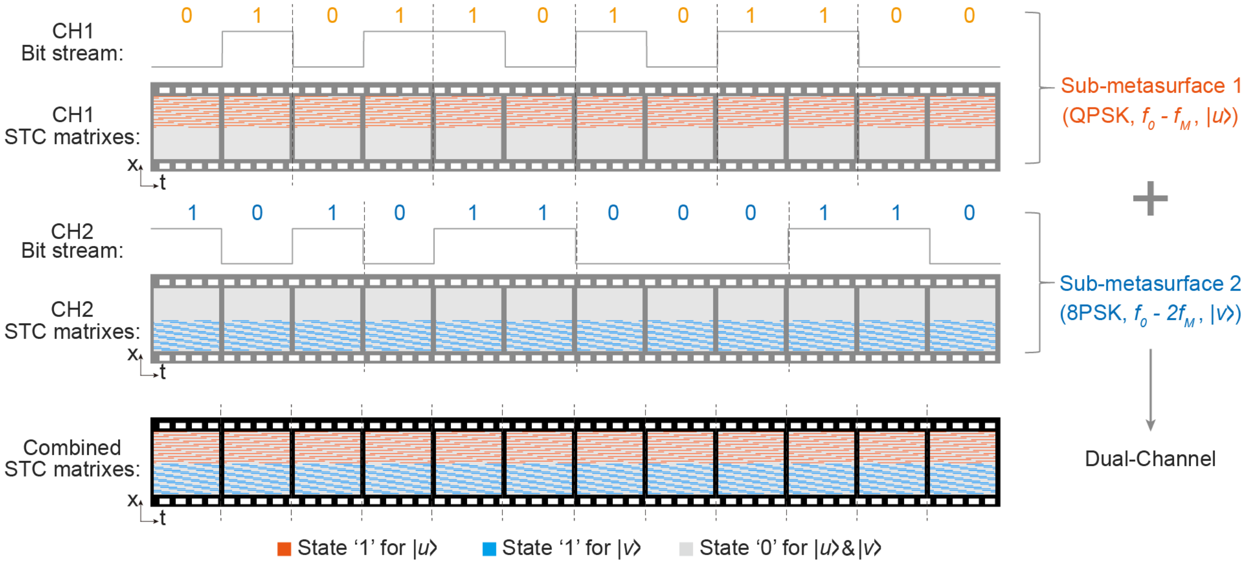

Supplementary Fig. S6 | The synthesis process of the dynamic STC matrixes for dual-channel wireless communications. The bit stream for each sub-metasurface is first mapped to the corresponding dynamic STC matrixes according to the desired conversion harmonic frequency, polarization, and signal modulation format. The final dynamic STC matrixes for dual-channel multiplexing is the sum of the two dynamic STC matrixes for the two interwoven sub-metasurfaces.

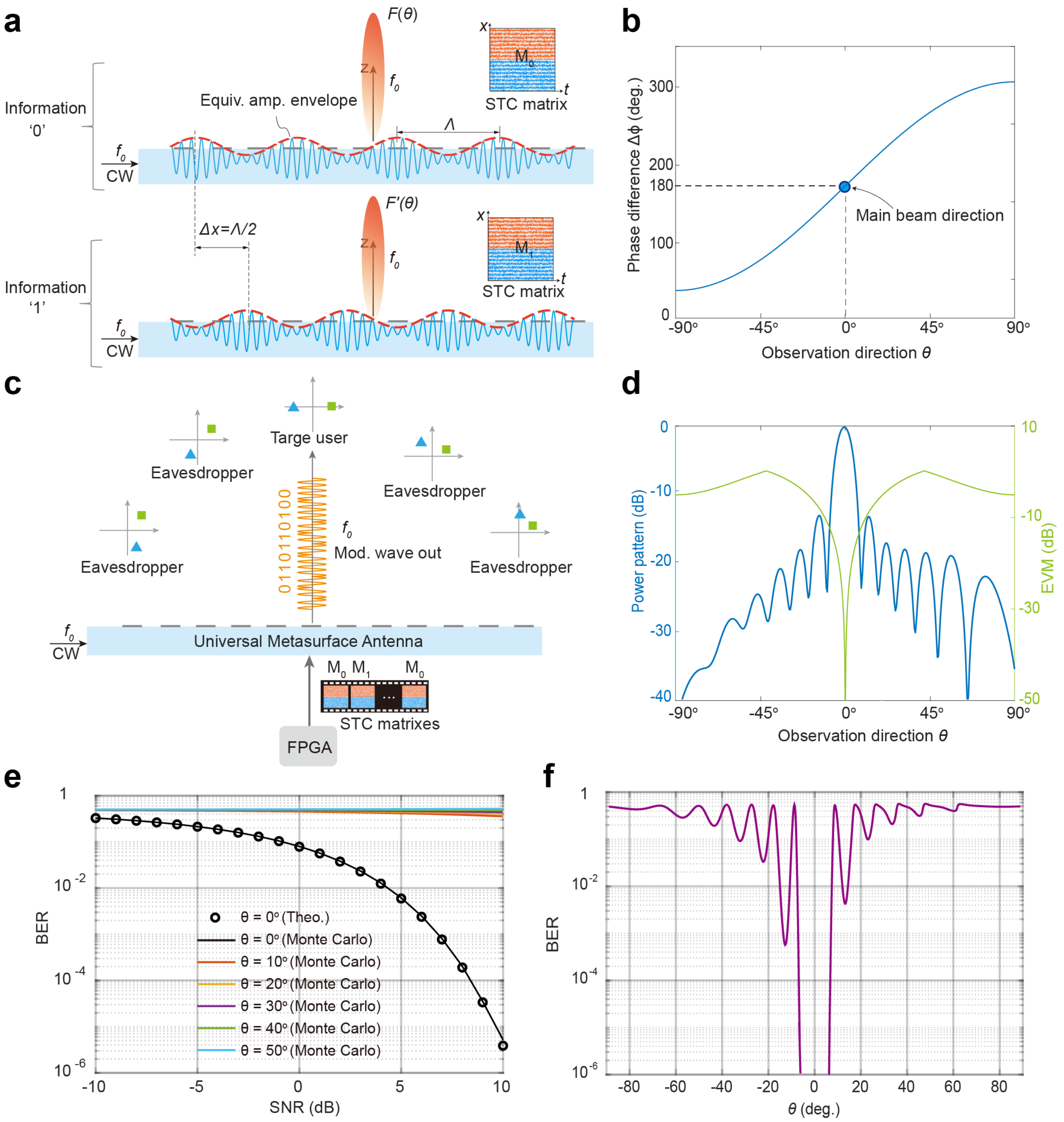

Supplementary Fig. S7 | The UMA with the inherent direction modulation for the BPSK scheme. a, The concept illustration of BPSK modulation by the UMA at the fundamental frequency. Two equivalent sinusoidal amplitude envelopes with a space shift of $\Delta x = \Lambda/2$ is achieved by the spatiotemporal modulation at the fundamental frequency. This results in high-directivity beams with 180° phase difference at the broadside direction. The right insets show the required STC matrixes for generating such spatial amplitude envelopes at the fundamental frequency. **b,** Phase difference between the two radiating cases as a function of the observation

direction θ. **c,** Concept illustration of the UMA for directly generating BPSK modulated waveforms at the fundamental frequency. Only the target user at the main-beam direction can receive the correct phase variation (information), whereas eavesdroppers at off-angles decode garbled constellations. **d,** Theoretical power pattern of the UMA and the EVM as a function of the observation direction. **e,** BER versus SNR for a receiver in different directions of the UMA. **f**, Calculated BER as a function of the observation direction based on the Monte Carlo simulation. The SNR at the main beam direction ($\theta = 0^o$) is set as 20 dB.

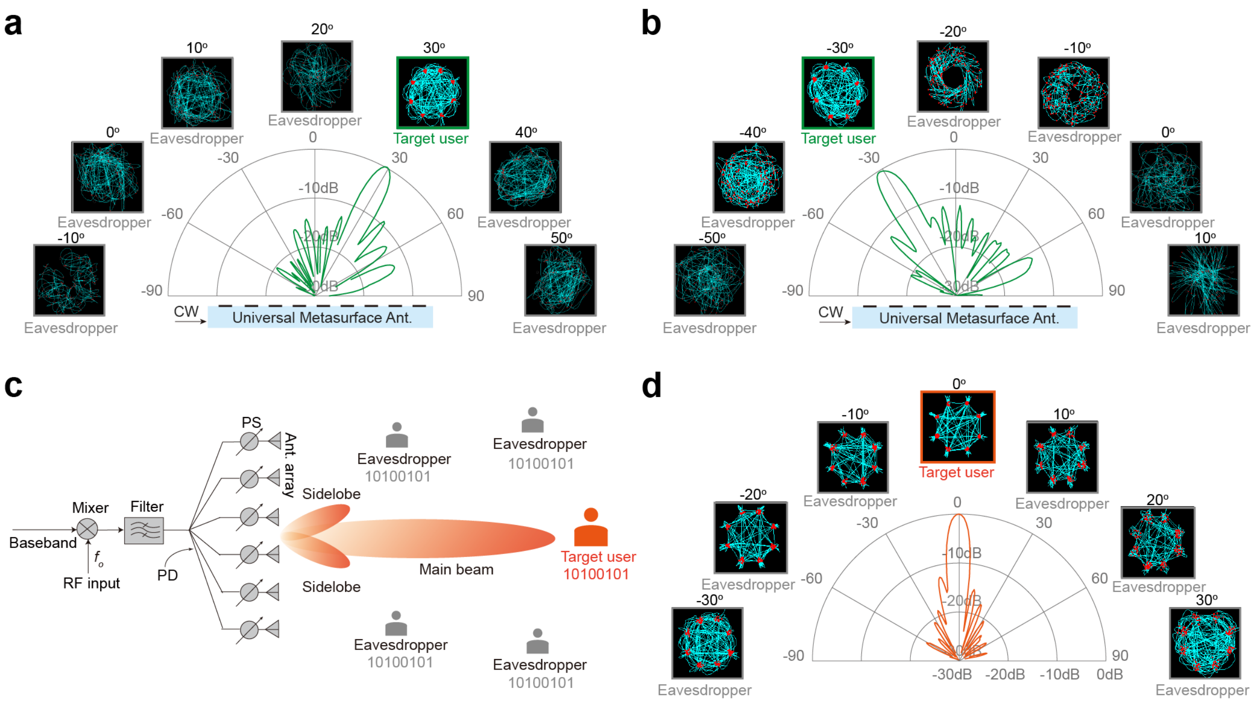

Supplementary Fig. S8 | More results on the physical-layer security communications using the UMA. a, b, Measured radiation patterns of the UMA at the fundamental frequency and the measured decoded constellation diagrams when the receiver is located in different directions. The main beam direction of the UMA is scanned to 30° (**a**) and –30° (**b**), respectively. Only the receiver in the main beam direction can successfully decode the information from the UMA. **c,** Concept illustration of conventional beam-scanning transmitter system, in which the free-space radiation carries the same time-varying wave properties (information) in all directions. **d,** The measured radiation patterns of the UMA operating at the $m = -1$ harmonic frequency, and the measured decoded constellation diagrams when the receiver is located in different directions. In this case, the receivers can successfully decode the information outside the main beam.

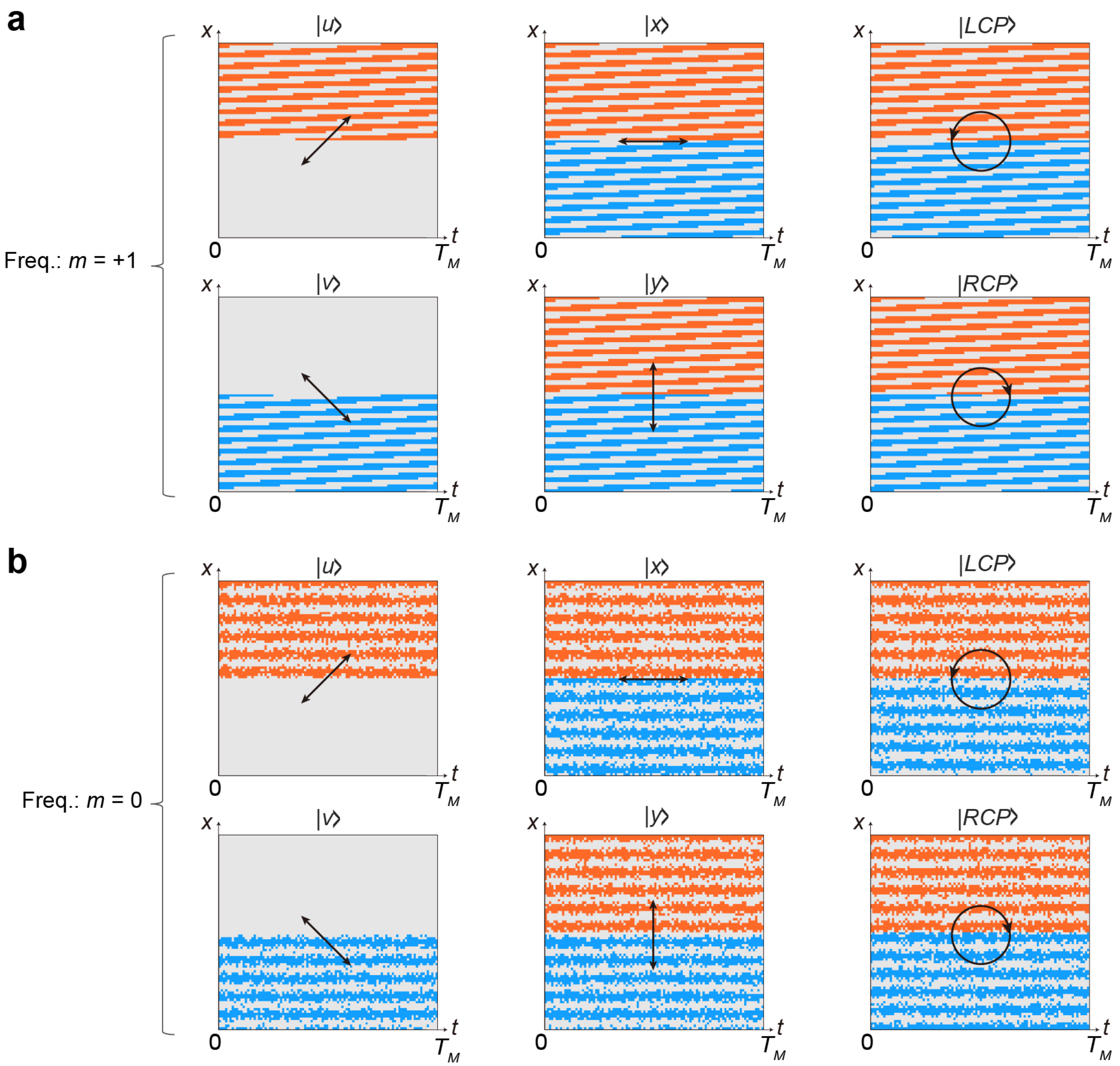

Supplementary Fig. S9| STC matrixes for realizing polarization control. a, The required '0/1' STC matrixes for polarization control at $m = +1$ harmonic frequency with different polarizations, including $|x\rangle$, $|y\rangle$, $|u\rangle$, $|v\rangle$, $|LCP\rangle$ and $|RCP\rangle$. **b,** The required '0/1' STC matrixes for polarization control at $m = 0$ fundamental frequency with different polarizations, including $|x\rangle$, $|y\rangle$, $|u\rangle$, $|v\rangle$, $|LCP\rangle$ and $|RCP\rangle$.

a
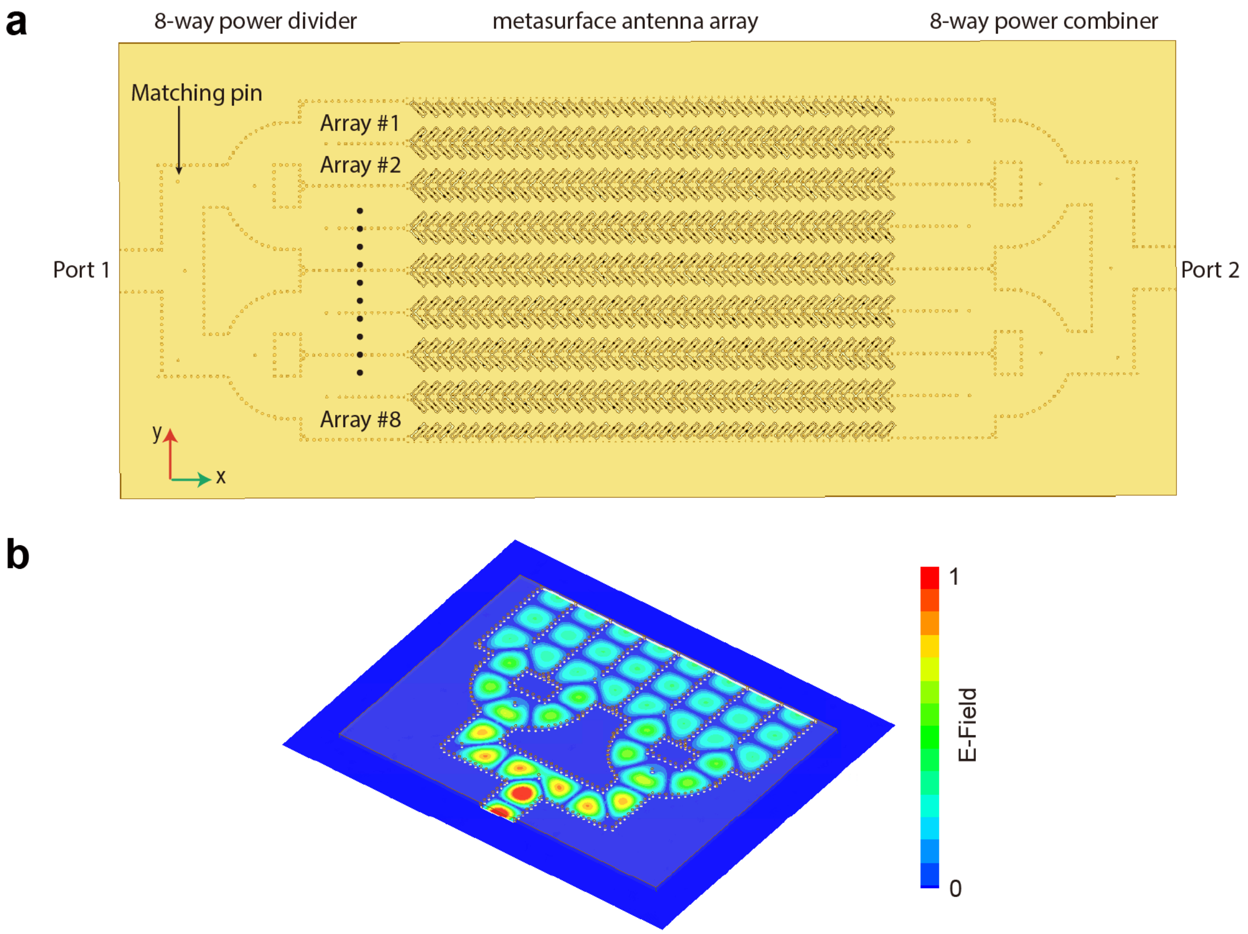

Supplementary Fig. S10 | a, Configuration of the 2D UMA. **b,** Simulated E-field distribution for the 8-way power divider at 23.5 GHz.

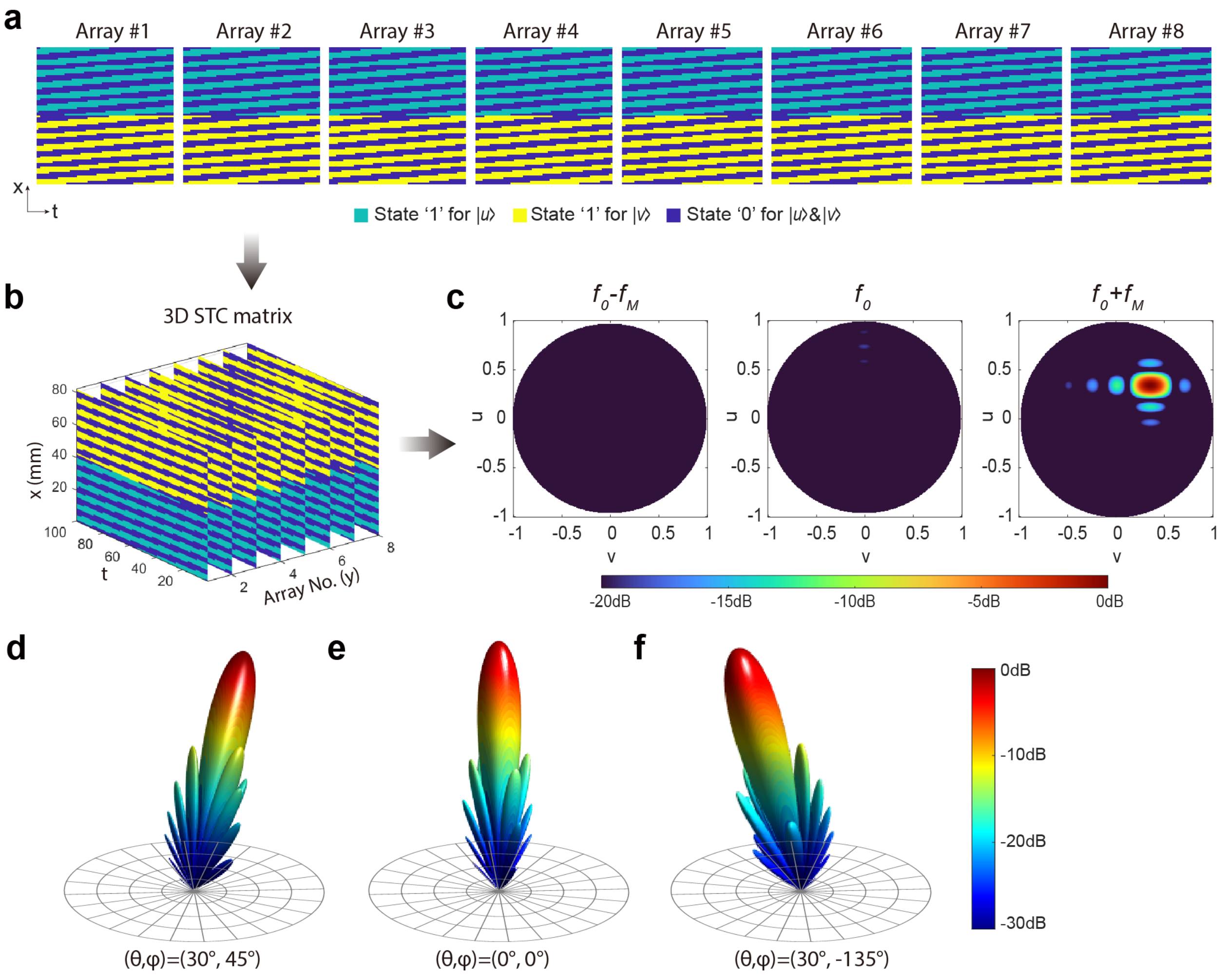

Supplementary Fig. S11 | 2D UMA for 2D wavefront engineering. **a**, 2-D STC matrix for each 1-D metasurface array to steer the beam to the direction $(\theta,\varphi) = (30°, 45°)$. **b-c**, The corresponding 3D STC matrix (b) and its calculated radiation patterns in uv-space at different harmonic frequencies (c). **d-f**, Calculated 3D radiation patterns at the 1st harmonic frequency as the main beam scans to $(\theta,\varphi) = (30°, 45°)$, $(0°, 0°)$ and $(30°, -135°)$, respectively.

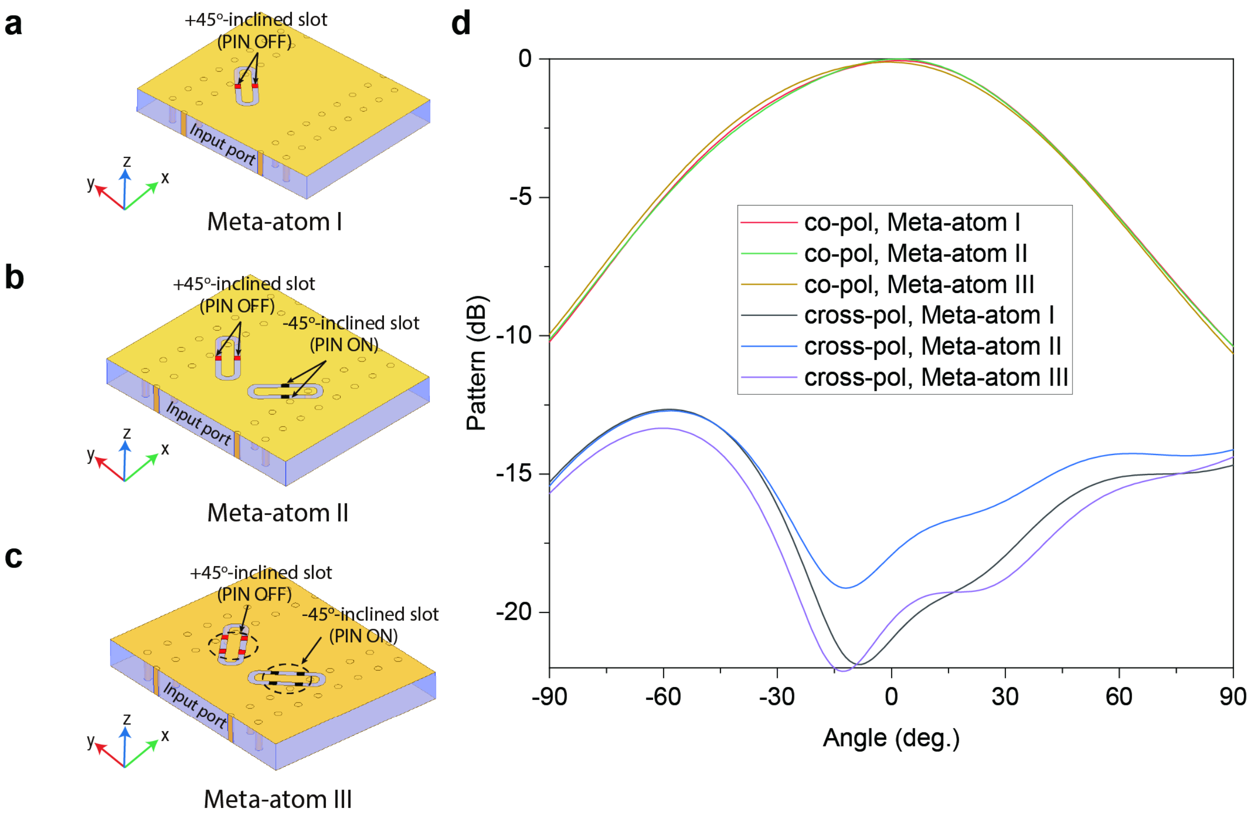

Supplementary Fig. S12 | Coupling effects of the two ±45°-inclined slot openings in each meta-atom. a, Meta-atom configuration with only one +45°-inclined slot opening on the top of the SIW waveguide. **b,** Meta-atom configuration with two ±45°-inclined slot openings. **c,** Meta-atom configuration with two ±45°-inclined slot-openings; each slot-opening has four PIN diodes to control its radiation state. **d,** Simulated co-pol (-45° linear polarization) and cross-pol (+45° linear polarization) radiation patterns for different meta-atom configurations.

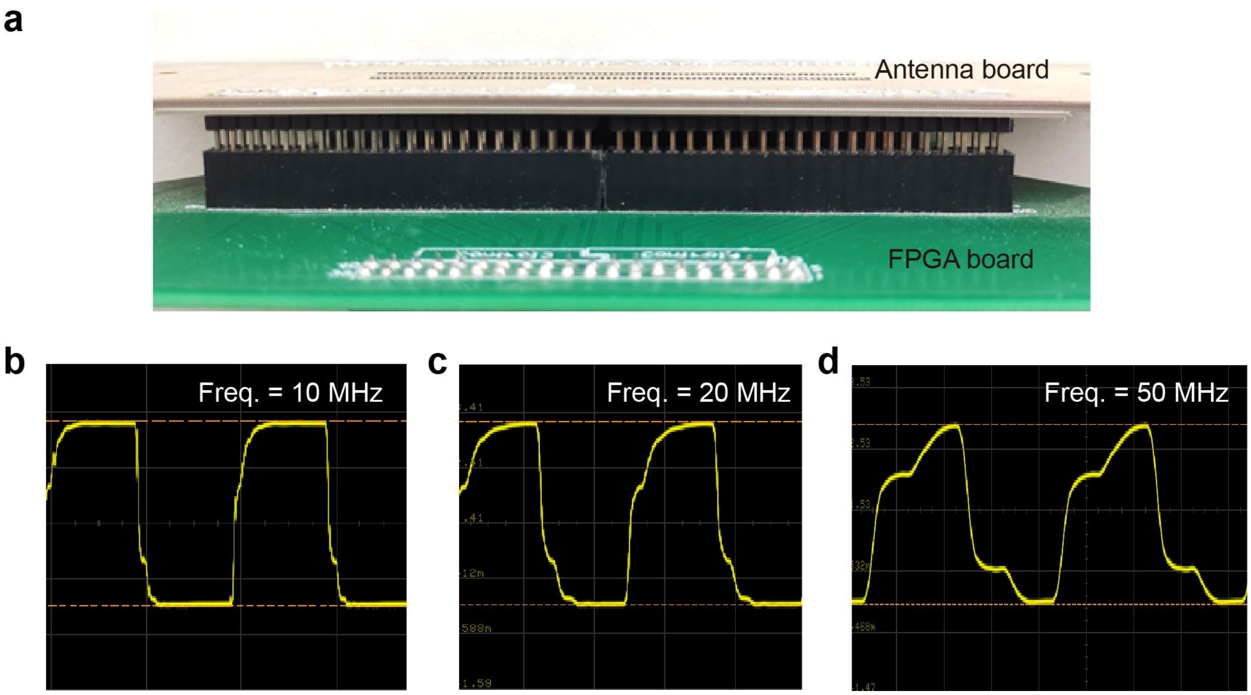

Supplementary Fig. S13. | Limitation for control signal. a, Photograph of the interconnect between the FPGA board and metasurface antenna board. **b-c**, Measured control signal waveforms from FPGA with frequencies of 10 MHz (b), 20 MHz (c), and 50 MHz (d) using an oscilloscope.

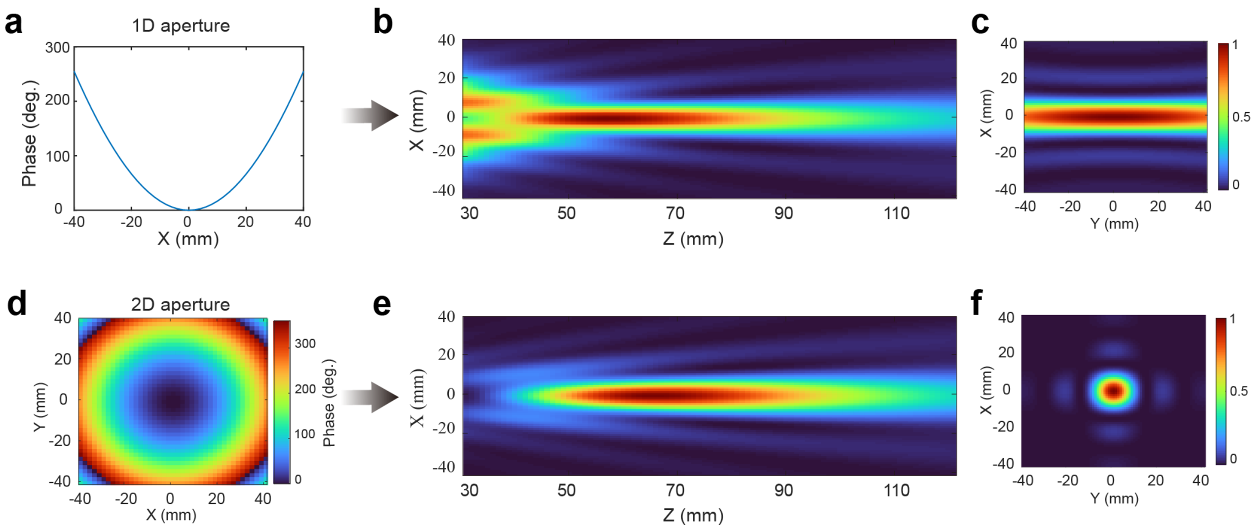

Supplementary Fig. S14 | 1D and 2D aperture effects on the fading distance. a-c, The aperture phase distribution for the 1D metasurface antenna (a) and its corresponding simulated E-field intensity distributions in the longitudinal plane (b) and transversal plane (c). **d-f**, The aperture phase distribution for the 2D metasurface antenna (d) and its corresponding simulated E-field intensity distributions in the longitudinal plane (e) and transversal plane (f).

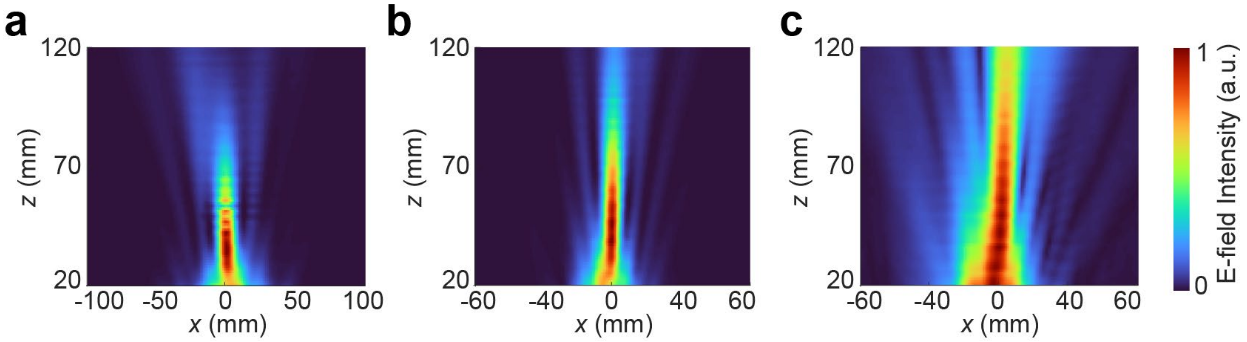

Supplementary Fig. S15 | Measured E-field intensity distributions at the $m = -1$ harmonic frequency for the designed focal point at $F(x_F, y_F, z_F)$ = (0, 0, 40) mm, (0, 0, 60) mm, and (0, 0, 80) mm, respectively.

Supplementary Table S1 | Theoretical and measured $|u\rangle$ and $|v\rangle$ components for the six representative polarizations

Polarization	$\|u\rangle$	$\|v\rangle$	$\|x\rangle$	$\|y\rangle$	$\|LCP\rangle$	$\|RCP\rangle$
Theo. $\|u\|/\|v\|$	∞	0	1	1	1	1
Theo. $\angle u - \angle v$	/	/	0°	180°	90°	-90°
m = +1 harmonic frequency						
Meas. $\|u\|/\|v\|$	10.4	0.12	0.99	0.95	0.92	1.02
Meas. $\angle u - \angle v$	38°	-21°	3.4°	177°	82°	-80°
Meas. PCR	99%	98.5%	99.9%	99.8%	99.3%	99.2%
m = 0 fundamental frequency						
Meas. $\|u\|/\|v\|$	16.1	0.09	0.89	1.52	0.90	1.11
Meas. $\angle u - \angle v$	10°	-26°	-6°	180°	93°	-102°
Meas. PCR	99.6%	99%	99.4%	95.8%	99.6%	98.6%

PCR: polarizaiton conversion ratio = $|E_{co}|^2/(|E_{co}|^2+|E_{cross}|^2)$

Supplementary References

1. Cheng, Y. J., Hong, W. & Wu, K. 94 GHz substrate integrated monopulse antenna array. *IEEE Trans. Antennas Propag.* **60**, 121-129 (2011).
2. Wu, Y. F., Cheng, Y. J. & Huang, Z. X. Ka-band near-field-focused 2-D steering antenna array with a focused Rotman lens. *IEEE Trans. Antennas Propag.* **66**, 5204-5213 (2018).
3. Wu, G.-B., Dai, J. Y., Cheng, Q., Cui, T. J. & Chan, C. H. Sideband-free space–time-coding metasurface antennas. *Nat. Electron.*, 1-12 (2022).
4. Goodman, J.W. *Introduction to Fourier Optics* (Roberts & Co. Publishers, Englewood, Colorado, 2005).
